# Comparison of the Human Bone Matrix Gelatin (HBMG) with Autogenous Bone Graft in Reconstruction of the Parietal Bone Defects in Rat: A Histological and Radiographic Study

**DOI:** 10.5681/joddd.2009.010

**Published:** 2009-06-05

**Authors:** Hossein Shahoon, Hamid Reza Azimi, Camellia Kianbakht

**Affiliations:** ^1^Assistant Professor and Head, Department of Oral and Maxillofacial Surgery, Shahed University of Medical Sciences, Tehran, Iran; ^2^Assistant Professor, Department of Oral and Maxillofacial Surgery, Shahed University of Medical Sciences, Tehran, Iran; ^3^Dentist, Private Practice, Tehran, Iran

**Keywords:** Autograft, bone defects, bone morphogenic protein (BMP), human bone matrix gelatin (HBMG)

## Abstract

**Background and aims:**

Autogenous bone graft is commonly used for reconstruction of bone defects in routine surgical procedures. The complexity of producing bone grafts and their application has lead to the use of human bone matrix gelatin (HBMG). The present study was conducted to compare the efficacy of HBMG and autograft on the reconstruction of bone defects in rats.

**Materials and methods:**

In this cross-sectional, experimental study, two defects were put on left and right sides of parietal bone of rats. HBMG was placed randomly on defects of one side and autograft in the defects of the other side. All specimens were assessed and compared with each other according to histological and radiographic characteristics. Other assessments included amount and the rate of bone formation, inflammation signs, fibrosis tissue and cartilage formation and also radio-graphic characteristics of grafts, assessed by digital and film-based methods. Mann-Whitney U test was used for statistical analysis.

**Results:**

The results showed a reduction of inflammation and an increase in new bone formation in both groups in 7, 14, 28 and 60 days after surgery. Bone formation with HBMG on day 24 was more than autograft. However, there was no sig-nificant difference between the groups on day 60. Superiority of digital method to film-based method of imaging was also observed.

**Conclusion:**

Although HBMG has the same efficacy as autograft, the rate of bone reconstruction with HBMG is higher. HBMG also induces focal, rather than peripheral, bone construction in the defect.

## Introduction

Bone tissue engineering is one of the most challenging concerns in today’s medical sciences research. Autogenic and allogenic grafts are two common methods of bone reconstruction; however, their use is associated with complications such as infection, pain, bone harvesting limitation, prolonged time of surgery, rejecting and a probable death ultimately. ^1,2^ For the past decades, many studies have experimented with suitable alternative materials for bone grafts. ^3,4^ Calcium phosphate derivatives have proved as appropriate substitutions for bone grafts. ^3-7^ Physical and chemical characteristics of these materials are very similar to natural minerals of bone, ^8^ and therefore, they result in less stimulation in the host tissues and less inflammatory reactions.

Restoration and regeneration of a tissue depend upon the proliferation and new matrix in injured area. Many authors have confirmed the successful defect reconstruction using demineralized bone matrix (DBM) or bone morphogenic gelatin (BMG), which contain many of bone constructing factors such as bone morphogenic protein (BMP). ^2,9^ Because DBM is a commercially available form of these materials, many studies have evaluated its bone reconstruction efficacy.

Investigators have reported successful results with BMG in animals; however, to avoid bioincompatibility, human bone matrix must be evaluated. Producing human bone matrix gelatin (HBMG) seems to be the next step for future studies to open a new horizon in the construction of bone defects. The aim of this study was the comparison of the efficacy of human bone matrix gelatin (HBMG) with autograft in the reconstruction of defects in parietal bone of rats.

## Materials and Methods

This was a cross-sectional, experimental study, performed in two stages in Shahed University of Medical Sciences and Tehran University of Medical Sciences. In the first stage, HBMG was produced from human humerus bone. Twelve mature male Sprage Dawley rats, 6–8 weeks of age, were selected. In the second stage, two defects were put on left and right sides of parietal bone of rats. Autografts from parietal bone of rat were also prepared. Both materials were put randomly in left and right defects. Data obtained from histological and radiographic evaluations of the experimented materials were collected on data sheets.

### First stage: Preparing human bone and producing HBMG

To produce BMG, we obtained a long bone with endochondral origin (humerus) from a bone bank (Imam Khomeini Hospital, Tehran, Iran). Specimens were assessed for conditions including age of donor, and absence of any systemic diseases such as diabetes, bone diseases like osteoporosis, and viral infections like HIV or HBS besides being a new bone (not protein-defect after donation or death). After screening laboratory tests were done, packages were preserved in a cold environment of −70°C.

Bone metaphysis was separated by a bone cutting bur. A cold water spray was used while cutting to prevent denaturizing of proteins. The bone shaft or diaphysis was cut and divided into 1–2 cm bone chips. Because of the thickness of human bone compared to animal model, more effective equipment had to be used. Bone chips of humerus were put in phosphate buffer for 24 hr and then remaining marrow, lipid and periosteum were scrubbed by brush. We used the method of Urist^2^ for producing HBMG from the prepared chips in six stages, as follows:

1. ** Chloroform- methanol solution with 1:1 proportion.**
Bone was cut in 2–3 cm pieces. The pieces were immediately put in chloroform methanol for 10 hrs in 25°C. This stage was intended for extraction of lipids as well as androgenic and controlling enzymes, which results in reducing dry weight of bone by 0.6 mg/g.
2. **Normal 0.6 chloridric acid**
In this stage, the bone pieces were put in a normal 0.6 acid chloridric solution to demineralize bone matrix and extract dissolved proteins in acid. The bone pieces remained in the solution for 30 hrs at 2°C. The solution was changed after 12 hrs. Dry weight of bone was reduced by 78-79% (bone powder).
3. **2 mol calcium chloride**
This solution was used to extract proteo-polysaccharides with low molecular weight in 2°C in a 30-hour period. The solution was changed after 12 hrs. Dry weight of matrix was reduced by 3.87% after this stage.
4. **0.5 mol EDTA solution**
0.5 mol EDTA solution was used in this stage to separate free calcium ions and extract phosphorylated proteins and proteopolysaccharides. This stage was done for 60 hrs at 2°C with a pH of 7.4 achieved by use of NaOH. Dry weight of matrix was reduced by 2.32%.
5. **8 mol lithium chloride solution**
8 mol of lithium chloride at 2°C was used for 30 hrs to compress collagen fibers and extract proteo-polysaccharides with high molecular weights, maintaining the pH of the solution at 5.5 by diluted chloridric acid. It should be noted that with high molarity of lithium chloride (up to 6), the temperature for collagen drying is reduced from 65 to 2°C. At the end of this stage, dry weight of the bone was reduced by 2.39% and the bones were compressed 40% in volume of bone collagen fibril. Electronic micrographs of collagen fibers prepared in linear cut showed lack or decrease of 640 Angstrom tropocollagen molecules, and also elimination of spaces which are naturally filled by fibril materials. As a result, collagen fibers had direct contact with each other.
6. ** 55°C distilled water**
Dissolved particles in bone matrix liquid were extracted in a 4-hour period. The weight of matrix was reduced by 16%.

### Preparing suitable sized particles of HBMG

After six stages, HBMG is mostly collagen-based and BMP is in its fibrum form. Since the size of HBMG is an important factor for bone and dentin construction, other procedures are necessary for transforming HBMG into a suitable size.

### Lyophilization

Lyophilization is the process of separating solid and liquid materials by freezing and drying in a vacuum environment. The lyophilization of HBMG pieces has two goals: (1) To ease drying and breaking of HBMG into pieces; (2) to separate water molecules from HBMG; this results in reducing enzyme reactions and destruction of HBMG. As a result, the lifetime of HBMG is increased followed by a high capability for bone construction.

### Chipping HBMG and sieving in suitable particles

To get a reasonable result with HBMG graft, particle sizes of 74 to 500 µm should be sized into 200 to 500 µm. Thus, after lyophilization, HBMG pieces were put in a mortar and liquid nitrogen was added to it and pieces of bone matrix were chipped by pestle. The next stage was the use of standard 250 and 500 micron sieves to separate suitable sized particles. Then an accurate scale was used to weigh HBMG in packages of 2 milligrams. HBMG was packed in aluminum plates and was conserved in −70°C until planted.

### Second stage: Operation for planting graft materials

In the operating room, xylozin (5 mg/Kg) was injected into dorsal muscle of subjects to achieve a general anesthesia and di-ethyl-ether to maintain it. First the operation site, the superior surface of skull, was prepped by iodine and was shaved. To access the posterior part of the head of rat, 2 cc 2% lidocaine with epinephrine was locally injected to control the bleeding and a 2 cm longitudinal cut was made by a No. 15 blade. The soft tissue and periosteum were retracted and two 5-mm deep cavities were created in left and right sides of parietal bone by a handpiece. The prepared grafts including bone pieces of parietal bone were put on one side as autograft, and the other side of the bone received 2 mg of HBMG, which filled the cavity.

The periosteum and flap were restored to their original position and the area was sutured with a 03 silk suture. The animal’s body was kept warm until regaining a complete consciousness. All rats were transferred in a separate cage to an animal room. They were fed on 5 mg/cc of 250 mg cephalexin in water and powder pallet (rat food) for 5 days and then it was replaced with solid pallet.

###  Histological evaluation

All specimens were fixed in 10% formalin and sent to pathology ward, Shahed University of Medical Sciences Faculty of Dentistry, to be evaluated histologically in. To decalcify the specimens, they were put in acid formic 10% for 10 days, and then washed with rising degree of alcohol for dehydration and then put in salicylate solution for clearance. To prepare tissue sections, they were blocked in paraffin and serial sections were prepared in 5 µm thickness. Prepared pieces were gathered by laboratory lamella covered with albumin. To dry pieces, they were conserved in 40°C for 12 hrs.

After adding dye to specimens, a pathologist evaluated them considering type of bone, amount of bone construction, fibrosis tissue and inflammation with light microscope with magnifications of × 40, ×100, and ×200. Digital photographs of specimens were prepared by a Light microscope (Olympus BX 41TF, Tokyo, Japan). Mann-Whitney U test was used for statistical analysis.

### Radiographic evaluation

Radiographic evaluation was done using film-based and direct digital methods to allow for comparison of the two techniques in this study.

## Results

###  Histological findings

**Day 7**

Seven days after planting HBMG and autograft, little amount of bone construction and no sign of calcification was observed. Inflammation and an increase in swallowed cells were found in three specimens cavities containing HBMG. Giant multi-nucleus cells, lymphocyte and monocyte cells were present. Signs of inflammation were also present around the cavities with autograft but to a lesser extent than around the cavities with HBMG. Fibrosis tissue around specimens with HBMG was less compared to those with autograft (Figure 1).


Figure 1. Histological view of autograft implant 7 days after surgery showed inflammatory cells around graft zone and bleeding around the cavity (a). Histological view of HBMG implant 7 days after surgery (b). Fibrosis tissue around HBMG particles can be seen.

**a:**
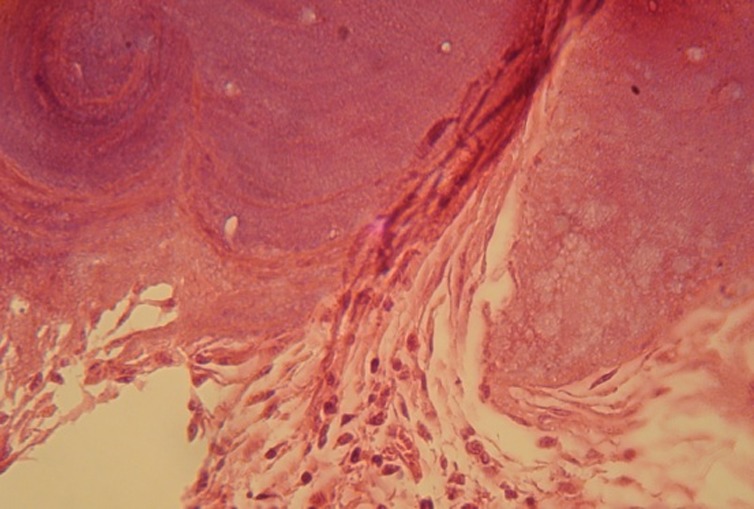

**b:**
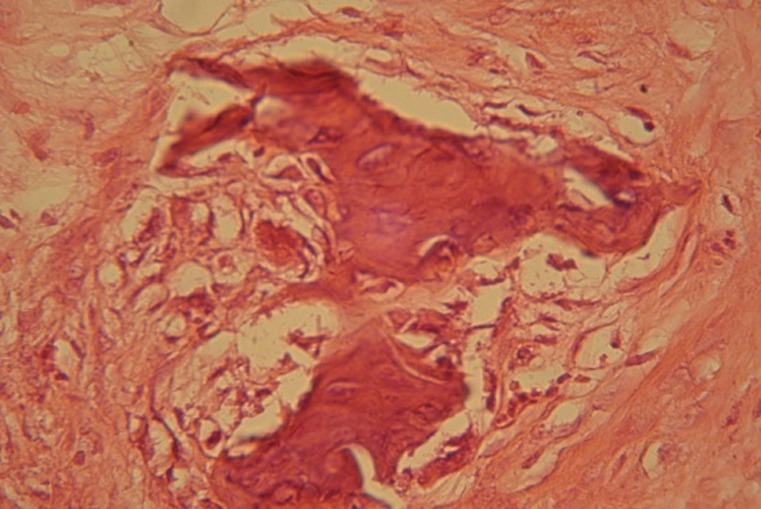

**Day 14**

Some grades of calcifications were observed on day 14. The calcification around specimens with HBMG was mostly in margin and center of cavities. The bone was immature and woven-like.

The amount of fibrosis tissue was reduced as the result of increase in bone construction. The specimens with autograft did not show evidence of fibrosis tissue. Inflammation in autografts of day 14 was reduced and there were no giant multi-nucleus cells around the cavities. Bone tissue of day 14 was a mixture of mature and immature bone, with the latter being dominant (Figure 2).


Figure 2- Histological section of autograft implants 14 days after surgery. Inflammatory cells also seen around autograft (a). Histological sample of HBMG implant 14 days after surgery (b). Cell differentiations around HBMG particles have involved more locations. Chronic inflammatory cells can be seen in this sample.

**a:**
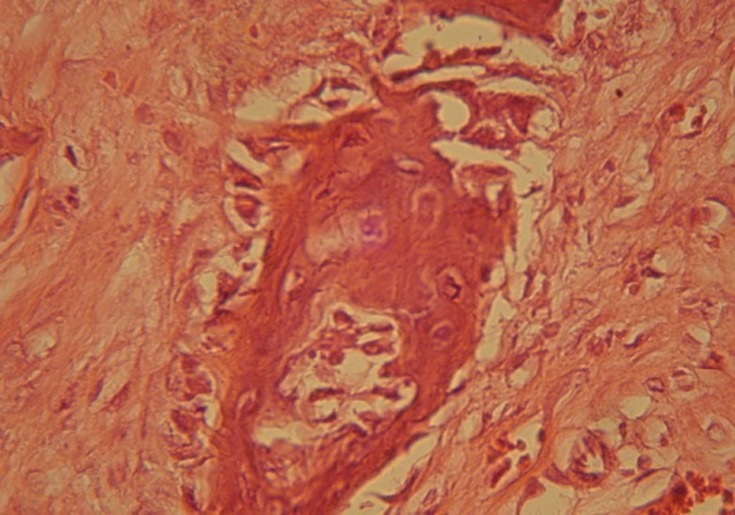

**b:**
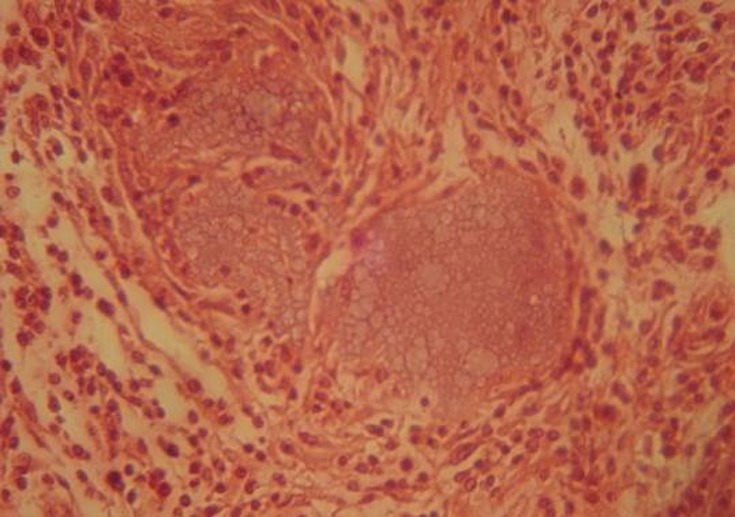

**Day 24**

The pathologist did not observe fibrosis tissue around the cavities neither with HBMG nor with autograft. Inflammation cells or tissue were not seen in the implantation area. The type of bone in day 24 was mostly intramembranous and the rate of bone construction in specimens with HBMG was more than autograft (Figure 3).


Figure 3. Osteogenesis, resorption and replacement of new bone on autograft can be seen (a). Lamellar bone formation at the site of HBMG implant is evident (b).

**a:**
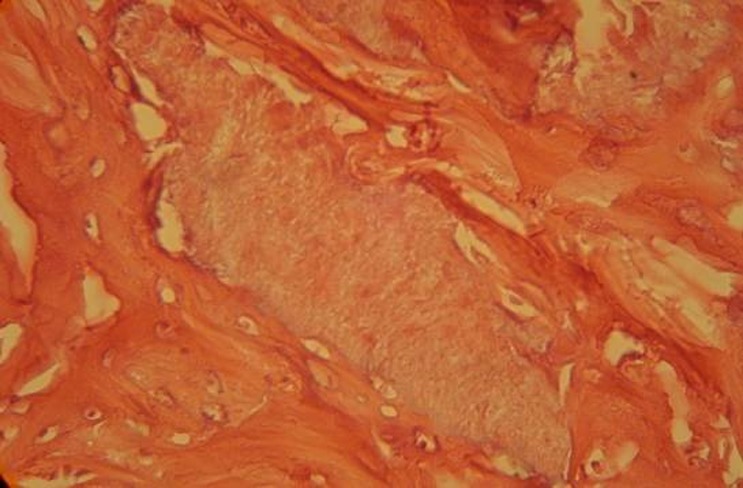

**b:**
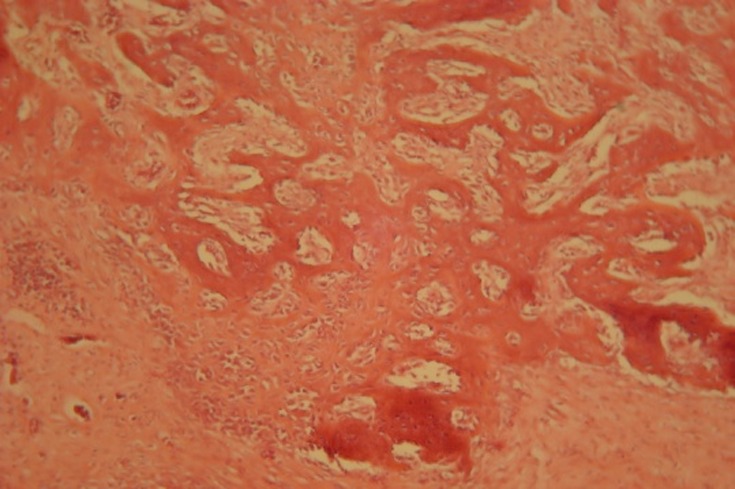

**Day 60**

Bone construction of day 60 advanced with mostly mature bones. New bone on day 60 showed a complete calcification very similar to the surrounding tissues; hence the cavities included mature and lamellar bone cells. Cavities with HBMG showed higher bone density and calcification compared with those containing autograft (Figure 4). No bacterial growth in ECBMG particles or cytotoxic effect on peripheral white blood cells were seen cultured on blood agar medium (Figure 5).


Figure 4. Using polarized light, mature lamellar bone and regular collagen bundles is clearly seen in HBMG site insertion on day 60 (a). Compact lamellar bone in ECBMG site is seen on day 60 in this section (b).

**a:**
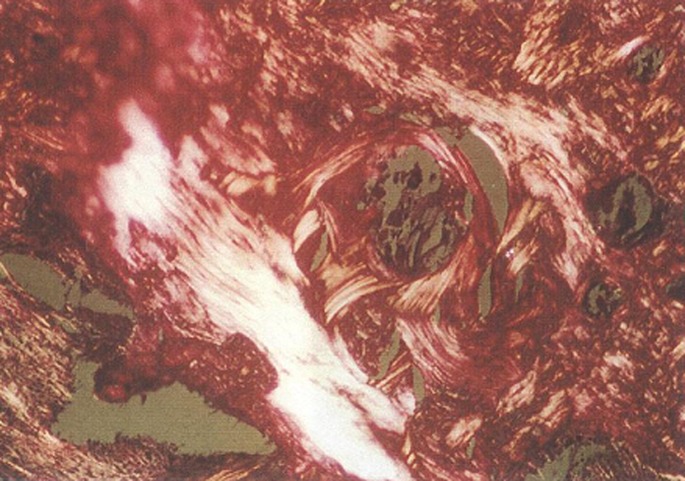

**b:**
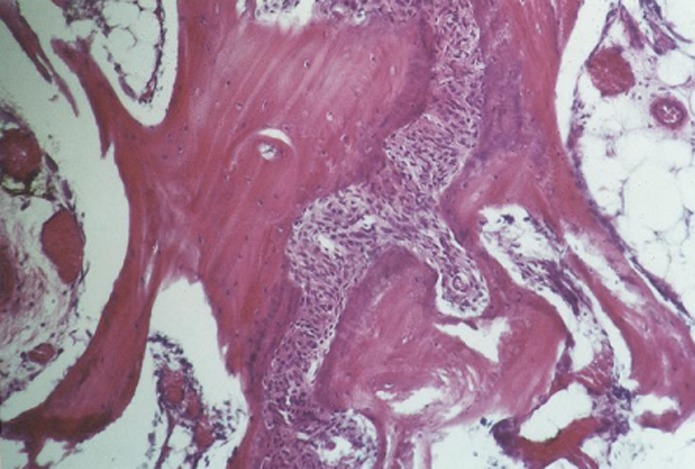


Figure 5. No bacterial growth in ECBMG particles in blood agar medium (A), and no cytotoxic effect on peripheral blood polymorphonuclear WBCs (B) was seen.

**a:**
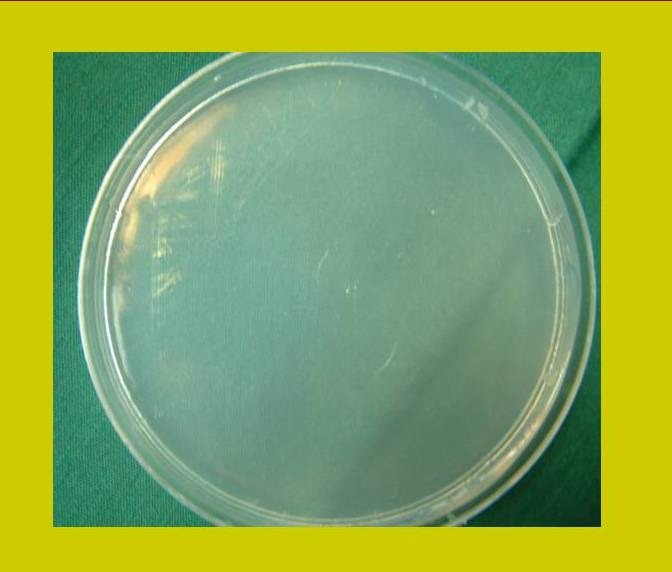

**b:**
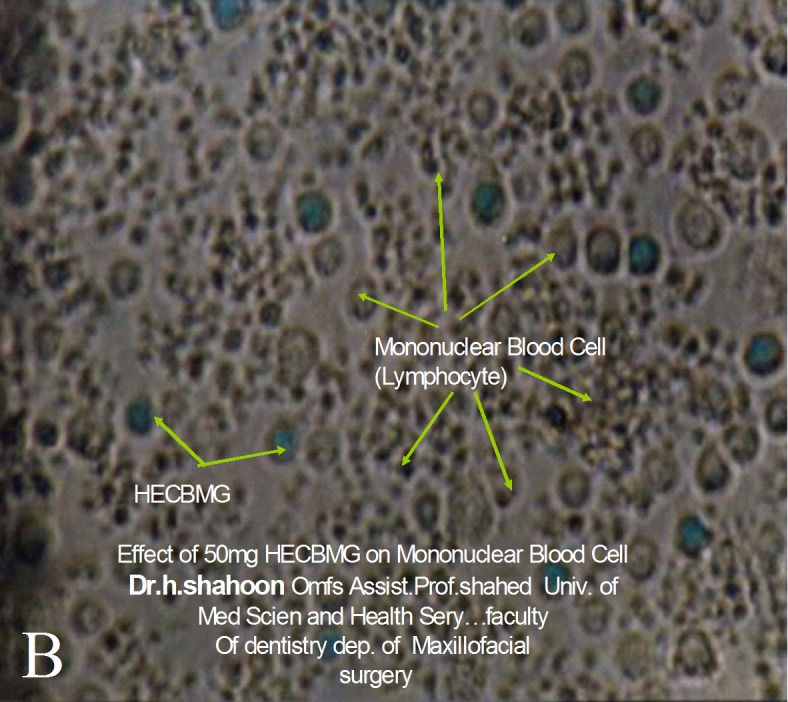

There were no significant differences between the efficacy of HBMG and autograft in bone construction in different days assessed in the study; however, HBMG showed a higher efficacy compared with autograft in a number of cases (Table 1).

**Table 1:** The results of histological evaluation of sections of each sample

		Type of new bone		Amount of new bone		Fibrosis tissue		Degree of inflammation	
Day	Case No.	HBMG	Autograft	HBMG	Autograft	HBMG	Autograft	HBMG	Autograft
7	1	-	-	+	++	+	++	-	+
	2	-	Woven	-	+	+	+	+++	+
	3	-	-	-	+	-	+	+++	+
14	4	Woven	Woven	++	+	+	+	+	+
	5	Woven Lamellar	Woven Lamellar	++	+	+	-	-	-
	6	Woven Lamellar	Woven	+++	++	-	-	-	-
24	7	Lamellar	Woven Lamellar	+++	++	-	-	-	-
	8	Lamellar	Woven Lamellar	+++	+++	-	-	-	-
	9	Lamellar	Lamellar	+++	++	-	-	-	-
60	10	Lamellar	Woven Lamellar	+++	++	-	-	-	-
	11	Lamellar	Lamellar	+++	+++	-	-	-	-
	12	Lamellar	Lamellar	+++	+++	-	-	-	-

-: zero; + low; ++: average; +++: high.

### Radiographic findings

Intra-observer and inter-observer viewer agreement in conventional film-based radiography were calculated as 64% and 72.9%, respectively.

For direct digital radiography, intra-observer viewer agreement was more than 80% (82% for observer 1 and 87% for observer 2) and inter-observer viewer agreement was 98%.

There was no significant difference between two bone substitutes in quantity of bone formation at the end of day 60 (P = 0.21). However, the new bone made in defects containing HBMG was significantly more opaque in comparison with bone made in defects containing autograft (P = 0.008). The differences between bone qualities were determined by digital images. Digital radiography was superior to film-based radiography in description of the mechanism and developing status of bone formation. Example of description of the pattern and shape of new bone formation with digital images are shown in Figure 6.


Figure 6. Radiographic evaluation 7 (a), 14 (b), 24 (c), and 60 (d) days after HBMG and autograft insertion.

**a:**
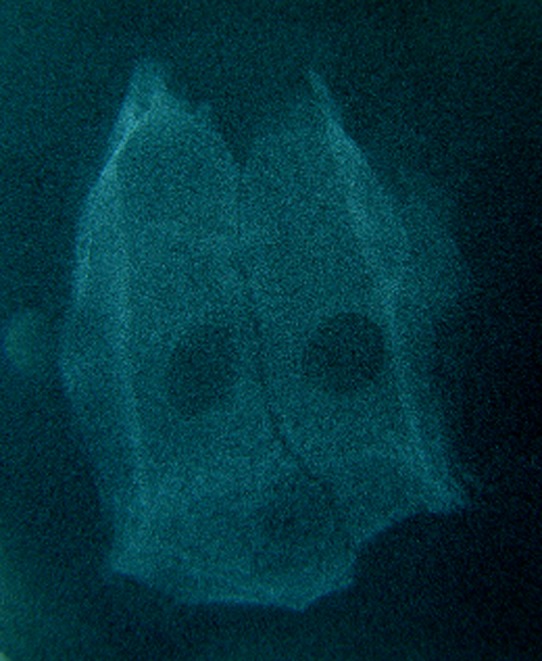

**b:**
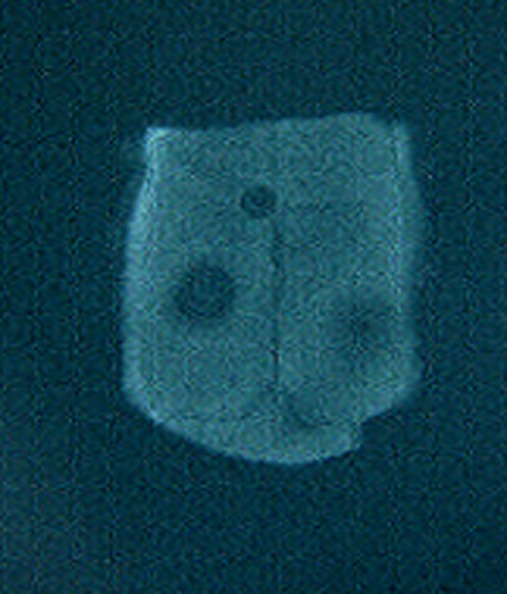

**c:**
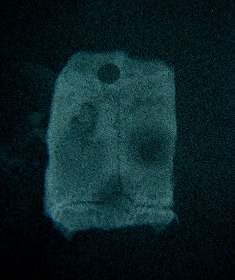

**d:**
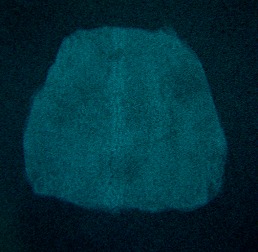

Comparisons of digital and film-based method are illustrated in Figure 7. Film-based and digital radiographic opacities of defects restored with HBMG and autograft in first and second weeks are presented in Tables 2 & 3. Agreement between autograft and HBMG and differences in radiographic opacity from day 7 to day 60 are shown in Figure 8.


Figure 7. Film-based (a,c) and digital (b,d) radiographs shown side by side.

**a:**
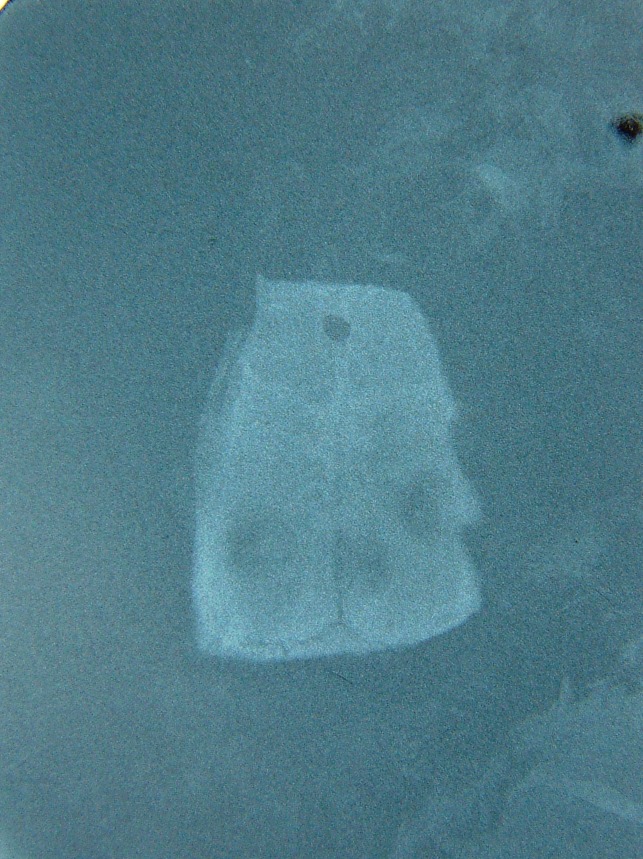

**b:**
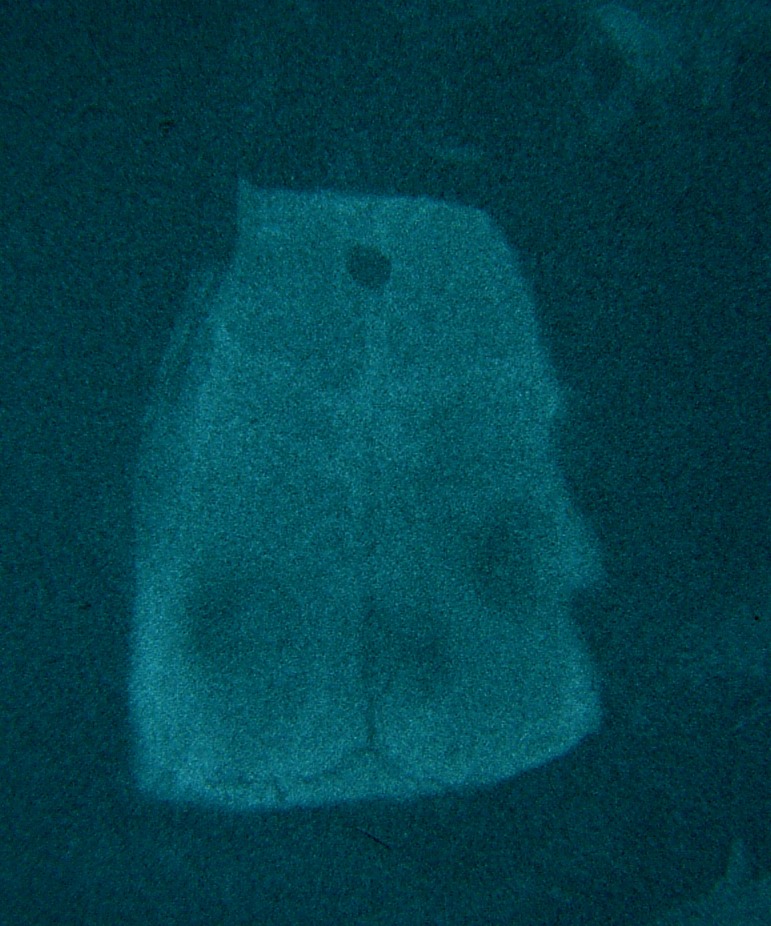

**c:**
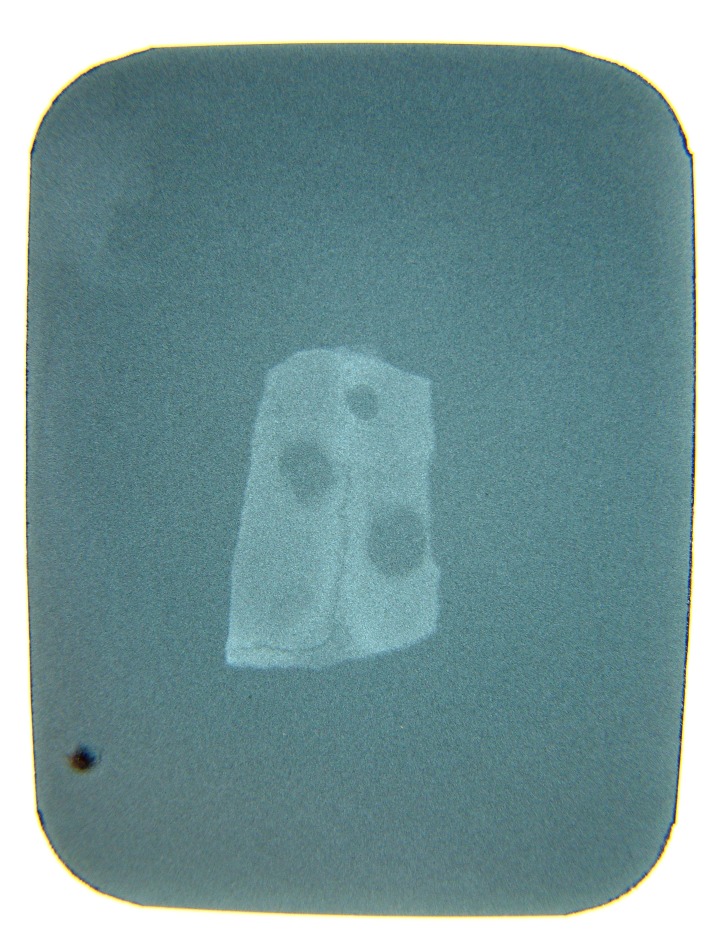

**d:**
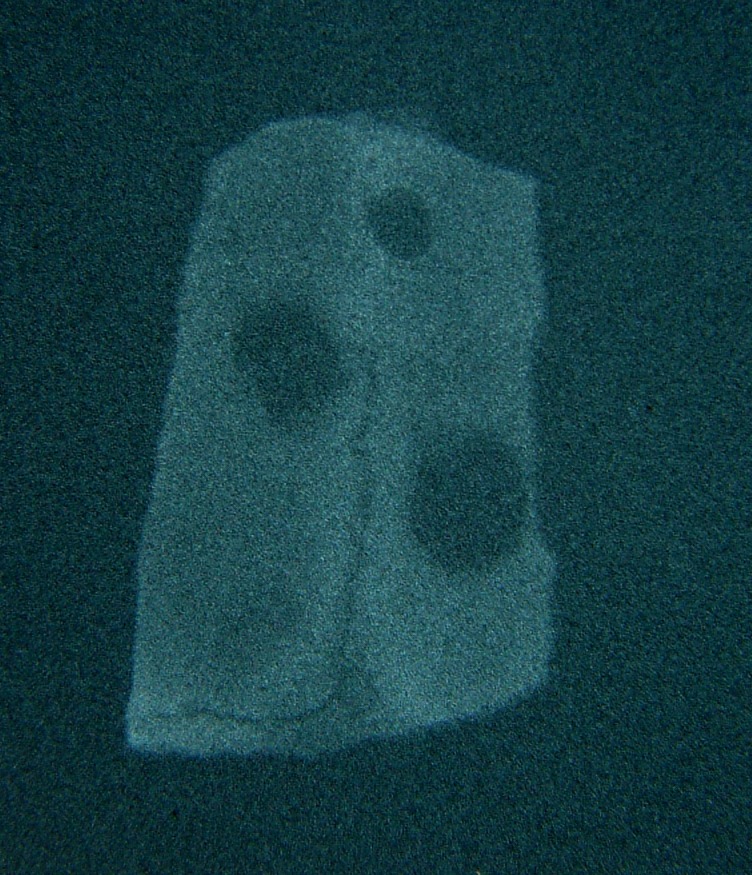


Figure 8. Agreement of radiographic opacity between autograft and HBMG in first (92%) and second week (74%) (left). No significant differences were observed in radiographic opacity between autograft and HBMG from day 7 to day 60 (right). (Opaque dominant (M.O); mixed, lucent dominant (M.L); completely opaque (C.O); and completely lucent (C.L))

**a:**
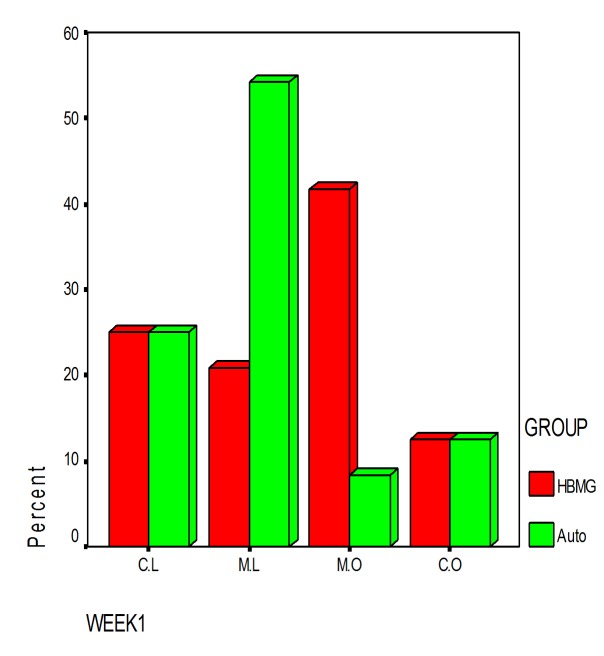

**b:**
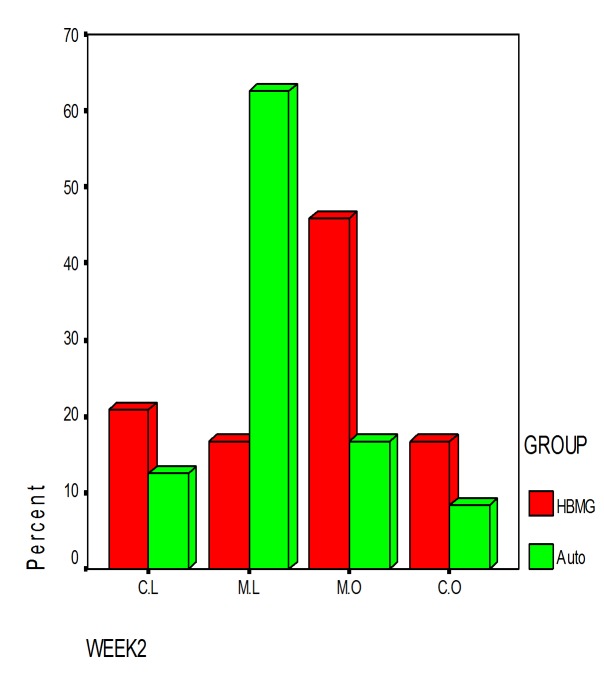

**c:**
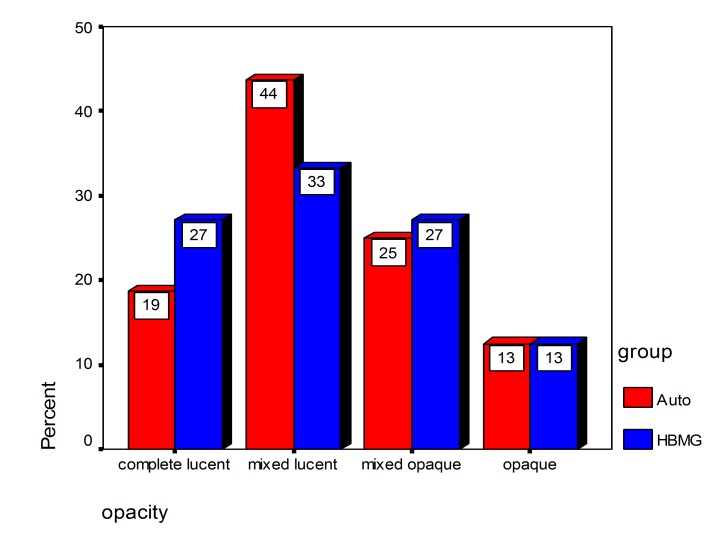

**Table 2:** Film-based radiographic opacity of defects restored with HBMG and autograft in first and second weeks

	Observer 1				Observer 2			
	First week		Second week		First week		Second week	
Case No.	HBMG	Autograft	HBMG	Autograft	HBMG	Autograft	HBMG	Autograft
1	C.L	C.L	C.L	M.L	C.L	C.L	C.L	M.L
2	C.L	C.L	C.L	M.L	M.L	M.L	M.L	M.L
3	C.L	C.L	C.L	C.L	C.L	C.L	C.L	C.L
4	M.L	M.L	M.O	M.L	C.L	M.L	M.L	M.L
5	M.O	M.L	M.O	M.L	M.O	M.L	M.O	M.L
6	M.L	C.L	M.L	C.L	M.O	M.L	M.L	M.L
7	M.L	M.L	M.O	M.L	M.O	M.L	M.O	M.L
8	M.O	M.L	M.O	M.L	M.O	M.L	M.O	M.L
9	M.O	M.L	M.O	M.O	M.L	M.L	M.O	M.L
10	M.O	M.O	M.O	M.O	M.O	M.L	M.O	M.L
11	C.O	C.O	C.O	M.O	M.O	M.O	C.O	M.O
12	C.O	C.O	C.O	C.O	C.O	C.O	C.O	C.O

M.O: Mixed, Opaque dominant; M.L: Mixed, Lucent dominant; C.O: Completely Opaque; C.L: Completely Lucent.

**Table 3:** Digital radiographic opacity of defects restored with HBMG and autograft in first and second weeks

	Observer 1				Observer 2			
	First week		Second week		First week		Second week	
Case No."	HBMG	Autograft	HBMG	Autograft	HBMG	Autograft	HBMG	Autograft
1	C.L	C.L	C.L	C.L	C.L	M.L	C.L	M.L
2	M.L	M.L	M.L	M.L	M.L	M.L	M.L	M.L
3	C.L	C.L	C.L	C.L	C.L	C.L	C.L	C.L
4	M.L	C.L	M.L	C.L	M.L	C.L	M.L	C.L
5	M.O	M.L	M.O	M.L	M.O	M.L	M.O	M.L
6	M.O	M.L	M.O	M.L	M.O	M.L	M.O	M.L
7	M.O	M.O	M.O	M.O	M.O	M.O	M.O	M.O
8	M.O	M.L	M.O	M.L	M.O	M.L	M.O	M.L
9	M.O	M.L	M.O	M.O	M.O	M.L	M.O	M.L
10	M.O	M.O	C.O	M.O	C.O	M.O	C.O	M.O
11	C.O	C.O	C.O	C.O	C.O	C.O	C.O	C.O
12	C.O	C.O	C.O	C.O	C.O	C.O	C.O	C.O

M.O: Mixed, Opaque dominant; M.L: Mixed, Lucent dominant; C.O: Completely Opaque; C.L: Completely Lucent.

## Discussion

The use of bone grafts have proved inevitable in various stages of health care and autograft is deemed the best kind among them; however, there are associated complications including donor limitation, prolonged period of improvement, suffering from extra surgery and probable death. This study was carried out on a human biomaterial to be used as an allograft, which prevents the complications associated with autografts. In the present study, two methods used for bone reconstruction using human bone matrix gelatine (HBMG) and autograft from parietal bone in rat were assessed.

The histological and radiographic assessment was accomplished in 7^th^, 14^th^, 24^th^ and 60^th^ day after operation. Since the human bone morphologic gelatin was not already produced in Iran and not used in previous studies, BMG of animals was used in their bone defects in other studies. BMGs used in previous studies were of rats,^10-12^ cats,^13^ rabbits,^11,14^ and dogs.^15^ To construct and restore human bone defects, a biomaterial of human body must be employed in order to suggest its application for future studies on human bone defects. In the present study, BMG was produced from humerus bone. Other authors have also used this type of human graft.^16-18^

Many authors have confirmed the construction properties of BMG and DBM and have concluded that osteoporosis reduces the efficiency of BMG in bone restoration. BMG, therefore, must be produced from a bone or be from a source without osteoporosis or any other bone diseases.^10^

Grafting with DBM has shown to be more successful than autograft in animal studies,^15^ while in the present study these two materials showed to have similar efficacies. This difference may be a result of the interval of evaluation of specimens. Bone construction was similar between cavities with HBMG and those containing autograft at the end of 60^th^ day, while the rate of bone construction from graft placement to day 24 was higher in cavities with HBMG.

In one study, bone calcification beside cartilage structure was observed in 7^th^ day with increase in the activity of alkaline phosphatase enzyme.^19^ Such a finding, however, was not seen in the present study. Bone construction from day 7 has been reported previously,^10^ which was also observed in the present study. The presence of giant multi-nucleus cells and inflammatory cells was observed in 7^th^ day in the specimens of this study, similar to the findings of a previous study.^20^

There were chronic inflammatory cells in both specimens with HBMG and autograft, while it had been reported there would be no immune reaction of host against HBMG.^16^

Findings of day 14 showed bone construction with mature and immature cells in both cavities; however, the calcification in cavities with HBMG was significantly more than cavities containing autograft. In another study on rat, 14 days after operation bone tissue was observed in the histopathological evaluation of defect and especially in its center, where HBMG was calcified.^21^ In the latter study, bone construction had cartilage structure while, in the present study, the construction was not intra-cartilaginous but rather intra-membranous, similar to a previous report.^10^ Since both studies were on the cranial bone of rat, and the graft material was BMG, the difference in the quality of bone construction may be a result of the type of BMG used, as in the present study it was produced from human bone.

In the present study, fibrosis tissue was observed in 7^th^ day in all specimens with autograft, which was reduced by the 14^th^ day. This finding for specimens with HBMG was the same while the cavities showed a little fibrosis tissue in 7^th^ and 14^th^ day after operation. Yamashita and Takagi^22^ reported a similar result.

Numerous studies have shown the two-phase intra-cartilaginous and osteochondral bone construction,^12,13,16,21-25^, while cartilage tissue was not observed in specimens with HBMG at any stages of the present study, which is similar to two previous reports.^10,26^

In the present study and other studies with non-compatible BMG,^10,11,26,27^ cartilage tissue was not observed in the cavities with BMG before calcification and the bone construction was intra-membranous.^28^ It has been suggested that using HBMG in cranial bones can affect both intra-cartilaginous and intra-membranous ossification with the latter being dominant, and hence, it can be used in skull bone defects.^10^

A hypothesis has been suggested that using HBMG in human may result in intra-cartilaginous bone construction, while in animals it may stimulate mesenchymal cells to differentiate to osteoblasts, and hence, the bone induction is intra-membranous.^10,26^ Reddi et al^29-32^ studied the whole process of bone construction including the reduction of fibrosis tissue and omitting the giant cells from 7^th^ to 27^th^ day, the results of which are similar to the findings of the present study; however, the presence of destroying of chondrocyte was evaluated specifically in the latter studies.

Histopathologic evaluation of specimens in 60^th^ day shows the existence of mature porous bone in the cavities with HBMG and autograft. Other studies have found the same findings in 60^th^ day.^10, 12-14, 16,17, 23,24,27,33-36^, Generally, however, HBMG is associated with fewer discomforts and complications compared with those associated with autograft, some of which include surgery for autograft, loss of blood during the operation, increased healing time, pain, and potential post-operation infection.

In radiographic evaluation of defects filled with HBMG during the healing period, the digital technique showed more benefits than conventional radiographs because of possibility of accurately defining image characteristics and reduced exposure to radiation. Using the digital technique, the diagnosis is more precise and closer to reality, confirmed by the histopathological evaluation.

## Conclusion

1. HBMG has only osteoinductive characteristics.
2. The rate of bone reconstruction using HBMG is higher than autograft and the quality is almost similar to normal tissue. In some cases, bone resulting from HBMG has a better quality in that the density of it is more than natural bone.
3. Osteoinduction with HBMG is either intra-membranous or endochondral depending on receiver of the graft.
4. HBMG has best biomaterial features among other graft materials.
5. HBMG is an antigen free material.
6. HBMG increases the focal bone construction and this prevents the bone from constructing peripherally around the cavity.
7. HBMG is absorbed during the bone construction, and hence, it replaces in original bone.
8. Our findings also demonstrated that digital imaging was superior to film-based technique for evaluation of bone formation using HBMG and autograft, some of which include the ability in picture enhancement and adjustment of exposure factors according to site of interest.
